# Unlocking Micro-Origami Energy Storage

**DOI:** 10.1021/acsaem.4c00702

**Published:** 2024-05-31

**Authors:** Wenlan Zhang, Hongmei Tang, Yaping Yan, Jiachen Ma, Letícia M. M. Ferro, Leandro Merces, Dmitriy D. Karnaushenko, Daniil Karnaushenko, Oliver G. Schmidt, Minshen Zhu

**Affiliations:** † Research Center for Materials, Architectures and Integration of Nanomembranes (MAIN), Chemnitz University of Technology, 09107 Chemnitz, Germany; ‡ Material Systems for Nanoelectronics, TU Chemnitz, 09107 Chemnitz, Germany; § School of Science, TU Dresden, 01069 Dresden, Germany

**Keywords:** micro-origami, energy storage, microbatteries, microsupercapacitors, monolithic integration

## Abstract

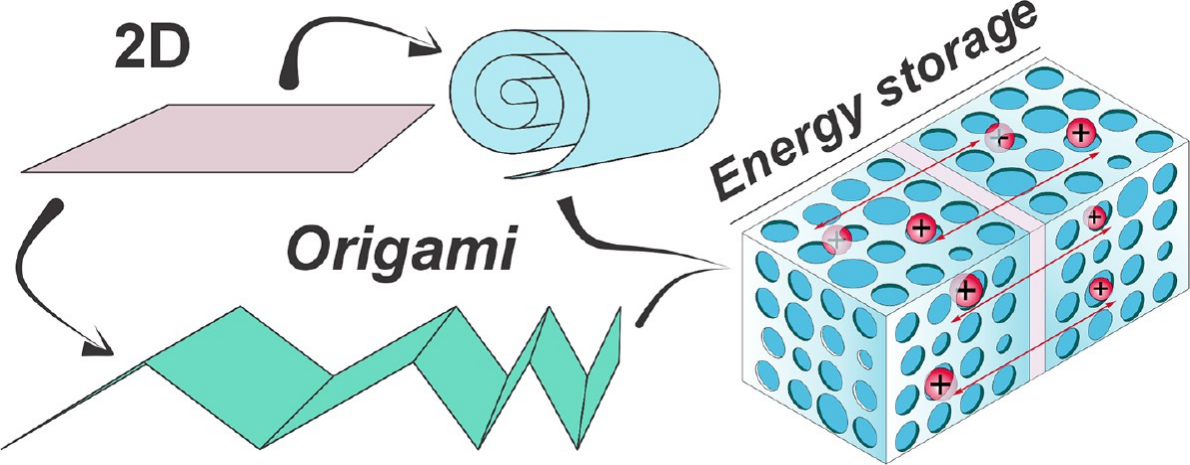

Transforming thin films into high-order stacks has proven effective for robust energy storage in macroscopic configurations like cylindrical, prismatic, and pouch cells. However, the lack of tools at the submillimeter scales has hindered the creation of similar high-order stacks for micro- and nanoscale energy storage devices, a critical step toward autonomous intelligent microsystems. This Spotlight on Applications article presents recent advancements in micro-origami technology, focusing on shaping nano/micrometer-thick films into three-dimensional architectures to achieve folded or rolled structures for microscale energy storage devices. Micro-Swiss-rolls, created through a roll-up process actuated by inherent strain in multiple layer stacks, have been employed to develop on-chip microbatteries and microsupercapacitors with superior performance compared to their planar counterparts. The technology allows additional functionalities to be integrated into the same device using multifunctional materials. Despite significant progress, the key challenge for micro-origami technology in creating microscale energy storage devices lies in diversifying shape-morphing mechanisms to expand material choices, improve process reliability, and enhance reproducibility. Additionally, developing a universal microscale energy storage device that can cater to various tiny devices is intricate. Therefore, considering the integration of energy storage into final applications during the development phase is crucial. Micro-origami energy storage systems are poised to significantly impact the future of autonomous tiny devices, such as smart dust and microrobots.

## Introduction

Adding more functions per unit area stands as the fundamental objective in the advancement of microscale intelligent devices, such as smart dust and microrobots. These microdevices, operating at submillimeter scales, hold immense potential for diverse applications from wearable plant sensors to swallowable surgeons.^1^ The challenge lies in efficiently integrating diverse functionalities into confined spaces. As for smart dust, the aim is to infuse tiny devices with sensing, computing, and communication capabilities, enabling them to operate collectively as a distributed sensor network.^2^ Similarly, in the domain of microrobots, the emphasis is on maximizing functionalities within compact structures, enhancing mobility, adaptability, and eventually, autonomy.^3^ To accomplish these feats, progress is essential not only in scaling electronic components but also in the miniaturization of on-board energy sources, including a critical focus on energy storage devices that untether microscale intelligent devices from both wired connections and wireless energy transfer.^4^ The autonomy facilitated by advanced energy solutions becomes instrumental in realizing seamless and self-sustained operations for these tiny yet powerful devices.

In the pursuit of developing batteries, a central concern is increasing the footprint energy density (i.e., energy per unit area). Given that the energy storage capacity correlates directly with material loading, a straightforward approach is increasing the thickness of electrode materials. However, this path faces inherent limitations because the thickness cannot be infinitely increased due to kinetic and mechanical constraints. As a result, the thickness of the electrode material is typically maintained below 100 μm, with a common value of 40 μm for an 18650 cell, for instance.^5^ Notably, as the area shrinks to the submillimeter scale, the challenges associated with kinetic and mechanical limits become more pronounced. Traditional wet processes adding conductive additives to the electrode material to alleviate kinetic limitations are not compatible with most available microfabrication techniques. A large aspect ratio—thickness significantly greater than the footprint—renders the film fragile, exacerbating the constraints on thickness. Consequently, these compounded limitations substantially decrease footprint energy density, rendering tiny batteries impractical. To effectively overcome the kinetic and mechanical barriers while accommodating more materials, a proven strategy is to layer materials sequentially, including electrode materials, separators, and current collectors, to create a cell stack. Cylindrical, prismatic, and pouch cells are all fabricated by winding or folding the cell stack multiple times.

The process of winding or folding can be carried out using tools like a mandrel with a diameter of 1 mm or larger. When downscaling, a hands-free technique, known as micro-origami, emerges as a viable alternative for winding or folding thin films. In this scaled-down approach, the folding and winding procedures can be performed in a high-throughput and parallel manner, presenting a notable advantage from a manufacturing perspective.^6,7^ Folds and windings seamlessly integrate with well-established planar lithographic techniques, such as photo-, e-beam, and nanoimprint lithography. In principle, any two-dimensional (2D) pattern, even those with nanometer-thick and complex details, can autonomously self-assemble into a three-dimensional (3D) structure. More importantly, micro-origami can be applied to a wide range of materials from metals to oxides and polymers.^8,9^ Thus, micro-origami holds considerable promise for microbattery development, especially for those with footprints below 1 mm^2^, while simultaneously enhancing energy storage per unit area. In contrast to previous reviews of the microbattery fabrication field, this article focuses on recent advancements using micro-origami technology to create energy storage devices at the microscale.

### Micro-Origami and Energy Storage

**Figure 1:**
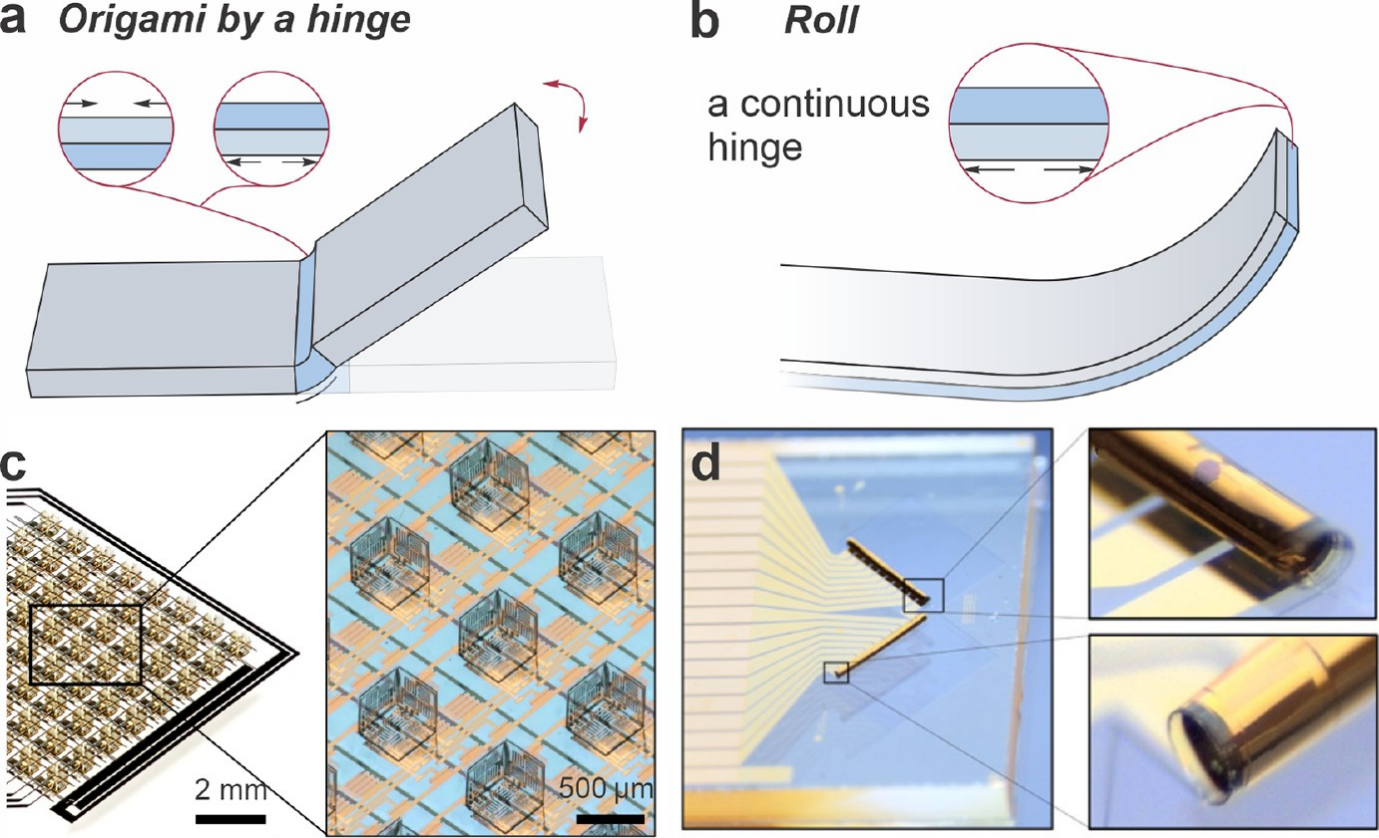
Folding and rolling. Schematics of (a) folding and (b) rolling. (c) An array of a cubic magnetic device created by the folding mechanism. Reproduced from ref (10). Available under a CC-BY 4.0 license. Copyright 2022 by the authors. (d) A Swiss-roll magnetic sensor created by the rolling mechanism. Reproduced from ref (11). Available under a CC-BY 4.0 license. Copyright 2019 by the authors.

Origami patterns transition from their deployed planar forms to folded states through the rotation of interconnected panels linked by hinges. The creases in origami act as hinges around which the panels rotate, allowing for various origami configurations to be achieved (Figure 1a). Starting with a compliant hinge design, several hinge actuation mechanisms come into play by incorporating active materials, resulting in the desired folding of a 3D origami structure. Whether it involves introducing a bilayer hinge with a contractible material on top, a bilayer hinge with an expandable material at the bottom, or an inactive hinge connected to active panels capable of generating torques, these approaches successfully achieve the general upward folding of the panels once the active material is actuated. Winding can be considered an extreme case of folding—a continuous hinge that rolls itself (Figure 1b).

Micro-origami incorporates microfabrication techniques for thin films, utilizing processes such as spin coating, photolithography, deposition, and etching to define geometries on a substrate. Subsequently, the planar thin film must be released from the substrate to transform into a 3D structure. To do so, a sacrificial layer should be applied as the bottom layer while enabling the fabrication of additional layers on top. Various options are available, ranging from metal layers like Cu,^12^ Al,^12^ and Ge^9,13,14^ to polymers such as poly(vinyl alcohol)^15^ and coordinated poly(acrylic acid) (PAA).^16^ Stimuli-responsive layers such as swellable hydrogels act as hinges. The swellable hydrogels normally contain polymeric backbones with ionic side groups.^17^ For anionic hydrogels, such as PAA^18^ and poly(methacrylic acid),^19^ when pH is larger than p*K*_a_ (acid dissociation constant), the side groups are ionized and generate fixed charges on the polymer, resulting in the volume expansion or contraction of the polymeric networks due to the electrostatic interaction and osmotic swelling force.^20^ For example, in neutral or basic conditions, carboxyl groups (−COOH) of PAA transform to a deprotonated form (COO^–^), causing gel swelling due to the electrostatic repulsion of COO^–^. When it is exposed to acid, COO^–^ protonation occurs, resulting in immediate gel contraction. Due to its deprotonation, PAA can restore its original state after the addition of a base.^21^ In contrast, cationic hydrogels, such as poly(dimethylaminoethyl methacrylate)^22^ and poly(2-(diethylamino)ethyl methacrylate)^23^ swell in acidic solutions (pH lower than their p*K*_a_). Such materials can be employed in photolithography by using their photocurability. Most of them are cast into a mold and polymerized under ultraviolet (UV) light, but precise on-chip control at a microscale was not explored. A mixed precursor consisting of N-(2-Hydroxyethyl)acrylamide and poly(ethylene-*alt*-maleic anhydride) can be precisely patterned by photolithography.^11,24,25^ This photopatternable hydrogel swells in an alkaline solution, generating force for micro-origami. Figure 1c shows an array of micro-origami self-assembled cubes fabricated with 3D magnetic vector field sensing ability, using the photopatternable hydrogel as hinges.^10^ When the photopatternable hydrogel extends to the entire bottom layer, rolled-up magnetic sensors were achieved (Figure 1d).^16^ The folding process is not limited to hydrogel actuation. The inherent stress within a multiple-layer material stack can trigger a self-rolling process, yielding diverse Swiss-roll materials, like SiGe, SiO_2_, TiO_2_, ZnO, Al_2_O_3_, C, Pt, SiN/Ag, Pd/Fe/Pd, and Au/Si/Ge.^26−30^

Some Swiss-roll materials have found application as electrode materials in lithium-ion batteries.^28,34,35^ The origami approach brings forth advantages such as maintaining a thin thickness and a hollow structure, addressing the kinetic and mechanical limitations inherent in electrode materials. Apart from being employed directly as active materials in an electrode slurry for full-sized batteries, a single Swiss roll can also function as an on-chip electrode for miniaturized batteries.^36^

**Figure 2:**
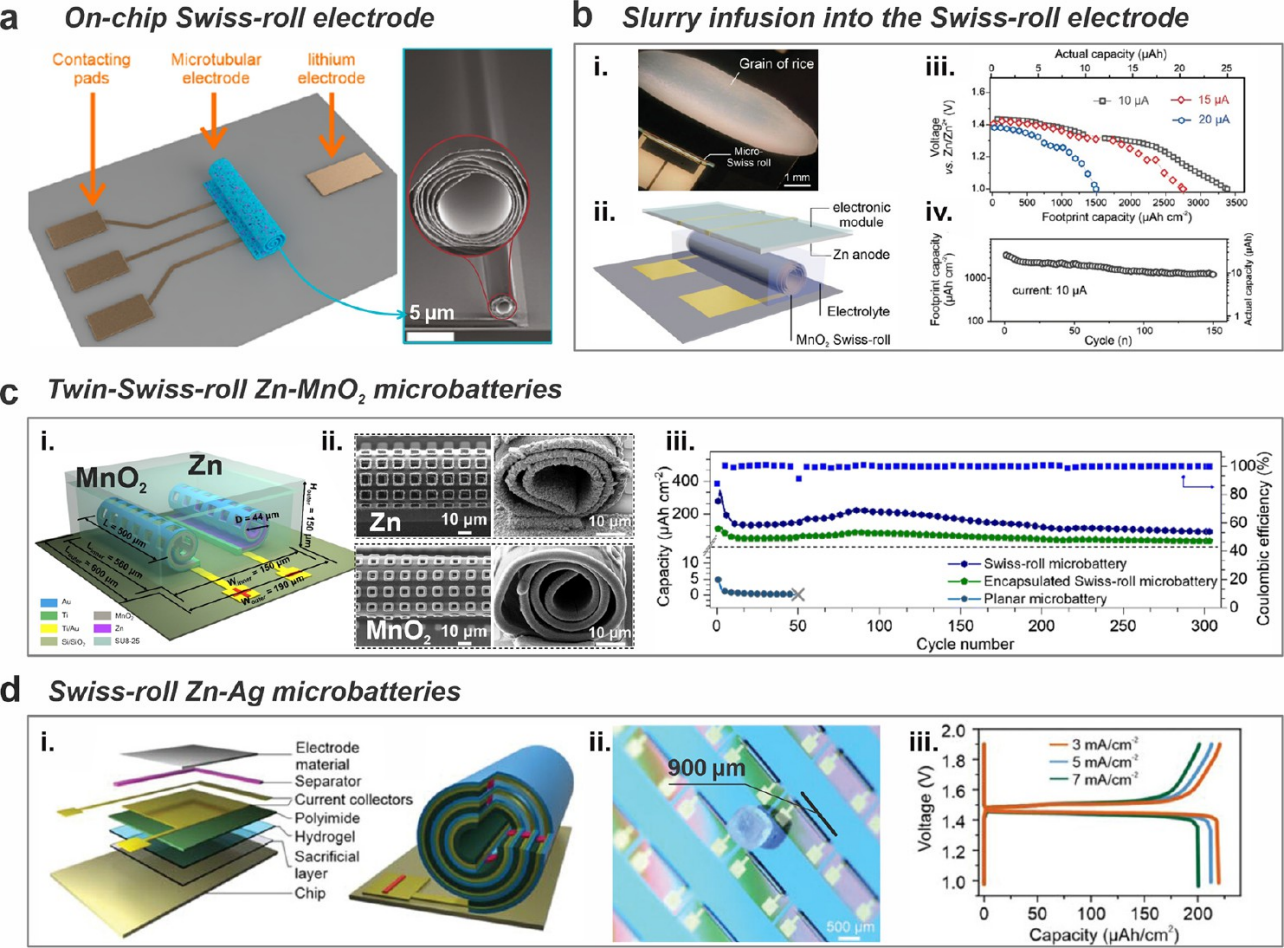
Swiss-roll microbatteries. (a) An on-chip micro-Swiss-roll Zn_2_GeO_4_ electrode for energy storage and in situ characterization. Adapted from ref (31). Copyright 2020, American Chemical Society. (b) A micro-Swiss-roll MnO_2_ electrode. (i) Image of the micro-Swiss-roll electrode compared with a grain of rice. (ii) Schematic diagram of a microsystem with a built-in microbattery. (iii) Galvanostatic discharge curves at different currents. (iv) Cycling performance at 10 μA. Reproduced from ref (25). Available under a CC BY-NC 4.0 license. Copyright 2022 by the authors. (c) A twin-Swiss-roll Zn-MnO_2_ microbattery. (i) Schematic diagram of the Zn-MnO_2_ Swiss-roll microbattery. (ii) SEM images of electrodeposited Zn and MnO_2_ Swiss-roll microelectrodes. (iii) Cycling performance at a current density of 5 μA mm^–2^. Reproduced with permission from ref (32). Copyright 2023, Royal Society of Chemistry. (d) A Swiss-roll Zn–Ag microbattery. (i) Schematic illustration of the Swiss-roll battery. (ii) Optical image of a Swiss-roll battery array compared with a grain of salt. (iii) Galvanostatic charge–discharge curves at different current densities. Reproduced from ref (33). Available under a CC BY-NC 4.0 license. Copyright 2022 by the authors.

Figure 2a illustrates a Zn_2_GeO_4_ Swiss-roll electrode on a glass substrate, serving as the anode material for lithium-ion batteries. Beyond its role in energy storage, the on-chip Swiss-roll electrode facilitates in situ characterization of material changes, providing precise insights into phase changes and thereby guiding the optimization of materials for enhanced performance. The diverse material choices for the on-chip Swiss roll also enable the incorporation of advanced electrode slurries (Figure 2b). A Swiss-roll current collector was formed with a footprint of 0.75 mm^2^ (Figure 2b, i). Such a tiny current collector can be filled with MnO_2_ slurry. Coupling with a Zn electrode (Figure 2b, ii), the MnO_2_ Swiss-roll electrode delivered an impressive footprint capacity of up to 3300 μAh cm^–2^ (Figure 2b, iii). Moreover, it maintained a reversible capacity exceeding 1000 μAh cm^–2^ across 150 cycles (Figure 2b, iv).^25^ These promising outcomes demonstrate the effectiveness of utilizing the micro-origami approach to realize compact and energy-dense electrodes on a chip. Inherent strain in multiple metal layers due to the lattice mismatch can also be used as the driving force for the winding process. The differential strain in Ti/Au layers accumulated during the deposition process. After etching the bottom sacrificial layer, Ti/Au layers can roll up, forming a Swiss-roll structure.^32^ The packaged twin-Swiss-roll cell using MnO_2_ and Zn as electrodes occupied a footprint area of 0.11 mm^2^ (Figure 2c, i). The porous structure can be engineered on the planar layer before the self-rolling process to improve the contact between the electrode materials and electrolyte (Figure 2c, ii). The Zn-MnO_2_ battery attained a high capacity of 136 μAh cm^–2^ (Figure 2c, iii) and an energy density of 181 μWh cm^–2^.^32^ This pioneering work expands the realm of microbatteries to the deep-submillimeter scale, a milestone not achieved by other techniques. It is also feasible to integrate the anode and cathode in one micro-Swiss-roll structure. Figure 2d illustrates a Swiss-roll microbattery using Zn as the anode and Ag as the cathode. This microbattery, smaller than a grain of salt (<0.1 mm^2^, Figure 2d, ii), delivered a stable output with a flat discharge platform and a maximum capacity of approximately 220 μAh cm^–2^ at 5 mA cm^–2^ (Figure 2d, iii).^33^

It is worth noting that the hollow space in Swiss-roll structures is not merely wasted space. It can serve several functional purposes depending on how it is used. For example, the hollow core can act as a reservoir, accommodating electrolytes to support the overall performance of the microbatteries. Besides, the hollow core can assist in material absorption through capillary forces. This feature effectively helps absorb electrode material slurry, as shown in Figure 2b, facilitating the utilization of active material powders in microbatteries. Moreover, the small hollow structure provides potential space for integrating sensors or other components in microfluidic applications, which can enhance the functionality of the microbattery.

Owing to the high stability of Zn batteries in air and water, they are compatible with micro-origami technologies. Various cathode materials, like copper, nickel, polyaniline and air are available for micro-origami energy storage. However, micro-origami technologies will not solve the inherent irreversibility issues of Zn batteries, but even amplify challenges because any side reactions may destroy the micro-origami batteries. Moreover, not all effective strategies to address the irreversibility issues of Zn batteries are accessible for micro-origami technologies, posing an additional level of complexity to achieve high-performance microbatteries. Furthermore, the energy density of Zn batteries is limited due to the relatively low voltage of less than 2 V.

Improving energy and power density to expand the application scenarios of microbatteries is the next immediate step for micro-origami energy storage. One strategy is to explore high-voltage battery systems. Lithium and lithium-ion batteries face fabrication challenges due to their sensitivity to moisture and air during micro-origami fabrication. Sodium and potassium have similar challenges. Magnesium and aluminum are stable in the air; however, these multivalent batteries lack suitable electrolytes. From a materials perspective, every material in batteries must be capable of withstanding heat, chemicals, and mechanical strains during fabrication. Any battery system needs to meet the energy and power demands of its target applications. Material loading is an important factor that determines the energy density, in addition to the operation voltage of batteries. Various techniques like physical/chemical vapor deposition and electrochemical depositions are available to produce battery materials. Although physical/chemical vapor deposition can produce a dense and smooth layer of materials, only a limited range of electrode materials are compatible with such deposition processes. The electrochemical deposition generally has fewer limitations in material choices. The main challenge of material deposition is to achieve high mass loading of electrode materials. Thick films develop strain and, therefore, cracks that sacrifice battery stability. Therefore, it is necessary to explore suitable deposition processes for battery materials. Alternatively, extending the rolling length is another viable approach, such as the long-rolling capacitors using magnetic-field-assisted assembly.^37^ The Swiss-roll capacitor design remarkably compacted its size by 100-fold, implying that a 0.030 mm^2^ rolled-up electrode contains materials equal to the amount in a 3 mm^2^ planar film. Misalignment is bound to happen when there is no mechanism to ensure proper alignment. Magnetic field has proven efficacy in aligning the roll-up process. To fully unleash the potential of micro-origami rolling, external field-controlled rolling is indispensable. Challenges are associated with the integration of magnetic materials that will not interfere with energy storage and the battery layer stack has a correct strain for rolling.

**Figure 3:**
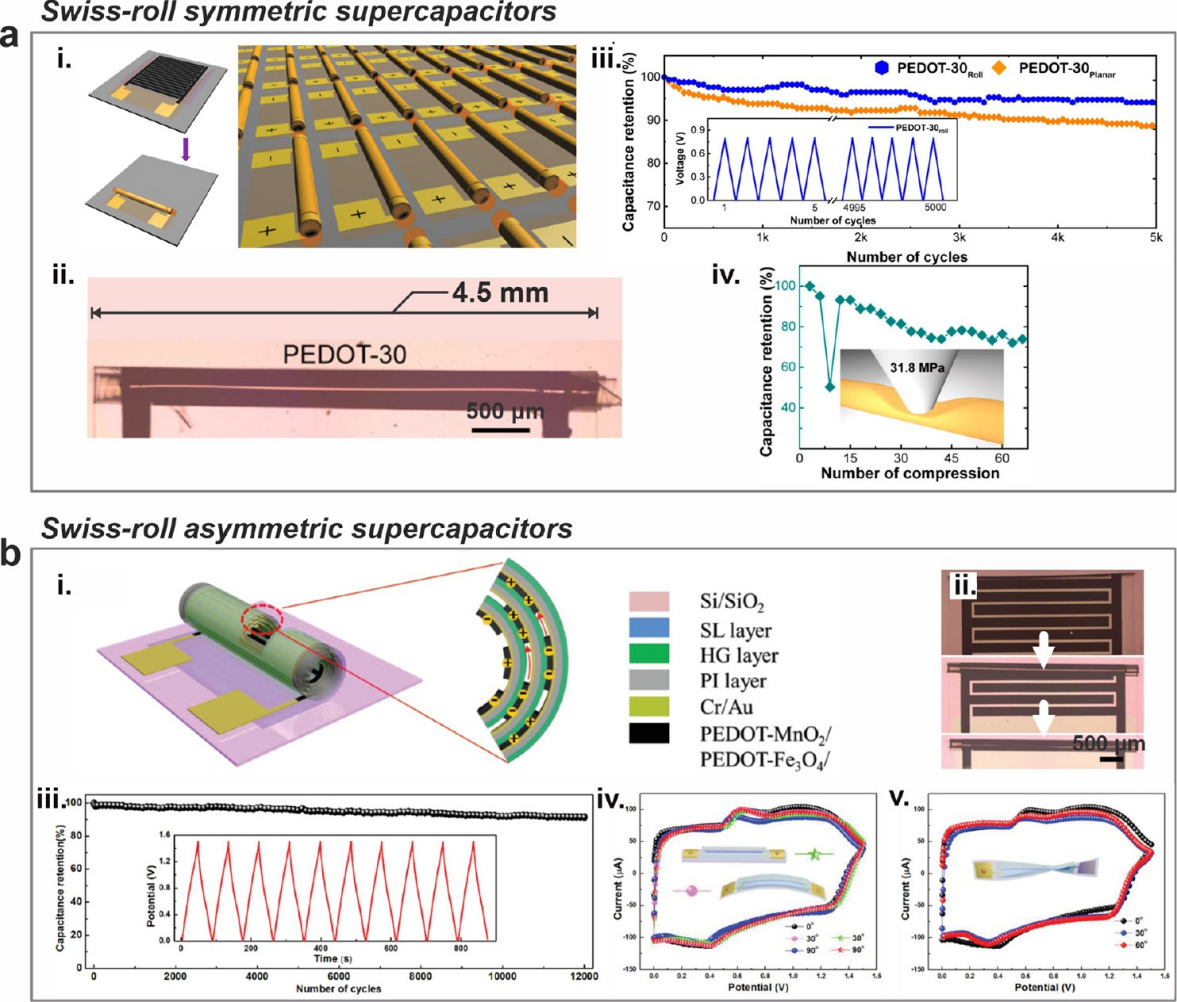
Swiss-roll microsupercapacitors. (a) A Swiss-roll symmetric microsupercapacitor. (i) Schematic illustration of the microsupercapacitor based on interdigital PEDOT electrodes. (ii) Image of Swiss-roll microsupercapacitor. (iii) Capacitance retention of Swiss-roll and planar supercapacitor. (iv) Capacitance retention of the Swiss-roll microsupercapacitor under compression. Reproduced from ref (38). Copyright 2019, American Chemical Society. (b) A Swiss-roll asymmetric microsupercapacitor. (i) Schematic illustration of the Swiss-roll microsupercapacitor. (ii) Images of self-rolling process. (iii) Capacitance retention of the Swiss-roll microsupercapacitor. Cyclic voltammetry curves at a scan rate of 200 mV s^–1^ when the Swiss-roll microsupercapacitor was (iv) bent and (v) twisted. Reproduced from ref (39). Available under a CC-BY 4.0 license. Copyright 2019 by the authors.

Supercapacitors share the same structure as batteries. Therefore, the micro-origami technique is also a feasible approach for developing Swiss-roll microsupercapacitors. For instance, an interdigitated electrode design for 2D microsupercapacitors can be directly layered atop the actuation polymer stack. Subsequent swelling during the etching process winds interdigitated electrodes up, eventually forming a micro-Swiss-roll supercapacitor. Figure 3a shows a micro-Swiss-roll supercapacitor with interdigitated electrodes made of poly(3,4-ethylenedioxythiophene) (PEDOT).^38^ After self-assembly, this Swiss-roll device occupied a small footprint area of approximately 1.5 mm^2^ (Figure 3a, ii), achieving an area capacitance of 82.5 mF cm^–2^ at a current density of 0.3 mA cm^–2^. Notably, the micro-origami supercapacitor demonstrates robust durability, maintaining up to 96.6% of its capacitance after 5000 cycles (Figure 3a, iii).^38^ Additionally, the device exhibited good resilience, effectively withstanding external compressive forces up to approximately 30 MPa, indicating its capability for self-protection under external stress (Figure 3a, iv).^38^ Diverse materials can be integrated in the Swiss-roll architecture. For example, an asymmetric supercapacitor using an electrodeposited PEDOT-MnO_2_ anode and a PEDOT-Fe_3_O_4_ cathode (Figure 3b) exhibited a large potential window (1.5 V) and large capacitance (88.6 mF cm^–2^ at 0.66 mA cm^–2^). As such, a high energy density of 28.69 mWh cm^–2^ at 0.25 mW cm^–2^ was attained. Additionally, the micro-Swiss-roll supercapacitor demonstrated long-term cycling stability, retaining 91.8% capacitance over 12,000 cycles (Figure 3b, iii).^39^ The micro-Swiss-roll supercapacitor can be bent and twisted without compromising its energy storage performance (Figure 3b, iv and v).^39^

**Figure 4:**
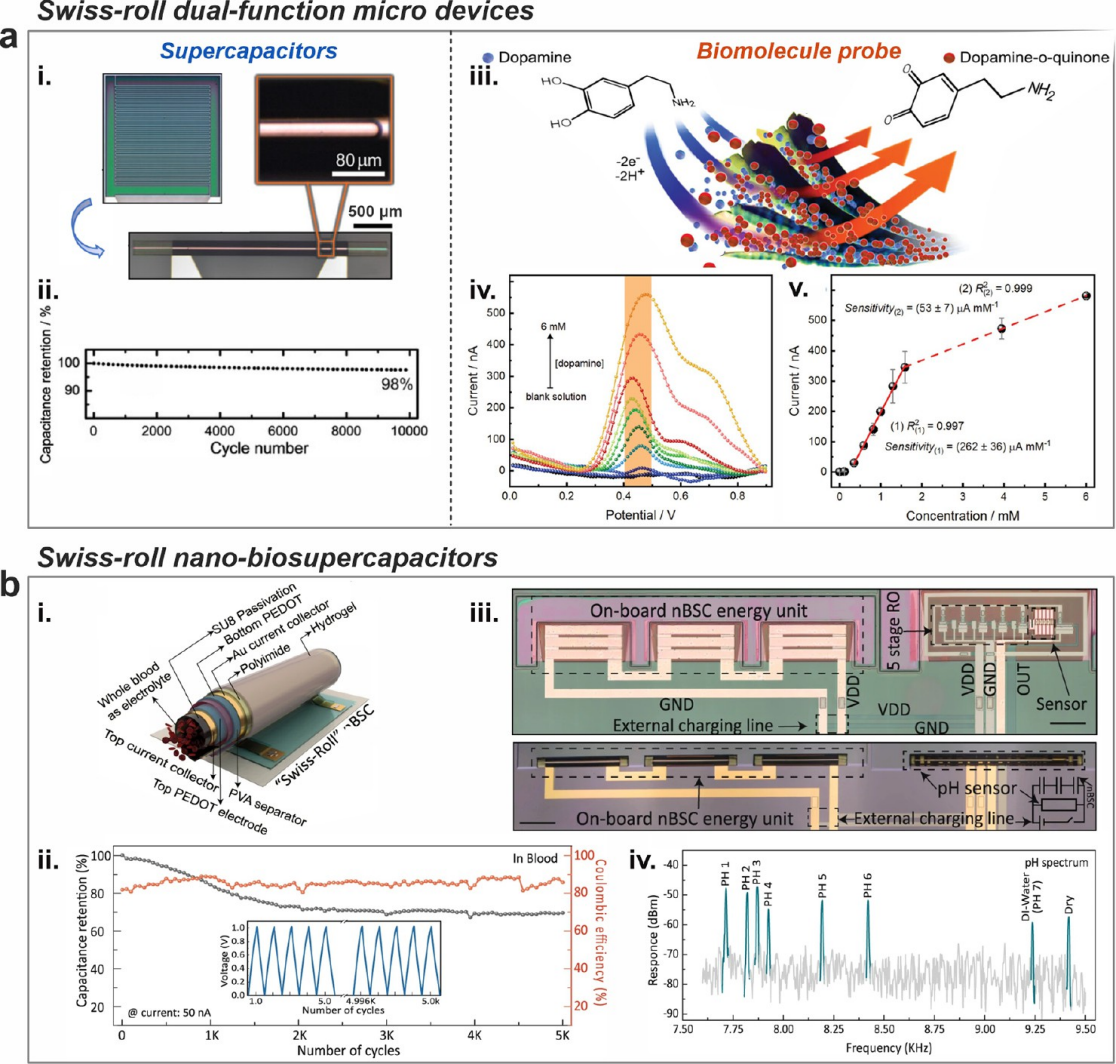
Multifunctional micro-Swiss-roll energy storage. (a) A dual-function micro-Swiss-roll device. (i) Optical images of the roll-up interdigitated electrodes. (ii) Capacitance retention over 10,000 cycles of the microsupercapacitor function. (iii) Sketch of the dopamine detection. (iv) Differential pulse voltammetry of the biomolecule probe function. (v) Calibration curves obtained from the peak currents of the transverse electrode as a function of dopamine concentration for the biomolecule probe function. Reproduced from ref (24). Available under a CC-BY 4.0 license. Copyright 2023 by the authors. (b) A Swiss-roll nanobiosupercapacitor (nBSC). (i) Schematic illustration of Swiss-roll nBSC. (ii) Capacitance retention of the Swiss-roll nBSC in blood over 5000 cycles. (iii) Microscope image of pH sensor with all integrated components before and after roll-up (Scale bars: 200 μm). (iv) Frequency spectral response of the nBSC based pH sensor. Reproduced from ref (40). Available under a CC-BY 4.0 license. Copyright 2021 by the authors.

Micro-origami energy storage systems are specifically engineered to provide power to various microsystems. Figure 4a presents a Swiss-roll micro-origami device (0.42 mm^2^) with dual functions, functioning as a supercapacitor and a biomolecule probe.^24^ As a supercapacitor, using electrodeposited PEDOT as the electrode material, it delivers a high power density (up to 911 mW cm^–2^) and good durability (over 10,000 cycles) (Figure 4a, ii).^24^ As a biomolecule probe, it also exhibited high sensitivity (230–262 μA mM^–1^) and selectivity in biomolecule detection, specifically dopamine (Figure 4a, iii-v).^24^ This micro-Swiss-roll device presents the opportunity to leverage redox electrochemical processes for creating versatile, multifunctional modules in microscale systems. Furthermore, a Swiss-roll nanobiosupercapacitor (nBSC) with a volume of just 1 nanoliter (1/1000 mm^3^) has been created, as illustrated in Figure 4b.^40^ This device is capable of delivering up to 1.6-V voltage in the blood (Figure 4b, ii) and its micro-Swiss-roll structure ensures efficient self-protection against the dynamic forces of pulsating blood or muscle contractions.^40^ At full capacity, it can power a sophisticated integrated sensor system to measure blood pH levels (Figure 4b, iii-iv). This breakthrough paves the way for the next generation of intravascular implants and microrobotic systems, enabling operation in small, difficult-to-access areas within the human body.^40^

### Open Challenges: Use Diverse Micro-Origami Mechanisms to Achieve Microfold and Micro-Swiss Roll

The integration of photopatternable and swellable hydrogel represents a noteworthy step in micro-origami for the development of micro-Swiss-roll energy storage devices with significantly improved footprint energy density. Nonetheless, the reliance on swelling within a solution imposes material limitations. For instance, metals are thermodynamically susceptible to corrosion in aqueous solutions. In addition, some polymer-based layers cannot withstand harsh working conditions, such as highly acidic or alkaline electrolytes, which depend on their intrinsic properties. For example, the polyimide and hydrogel layer will degrade in highly concentrated alkaline electrolytes. Exploring different polymer materials that can be patterned through photolithography and resist various working conditions is highly valuable for micro-origami technologies.

**Figure 5:**
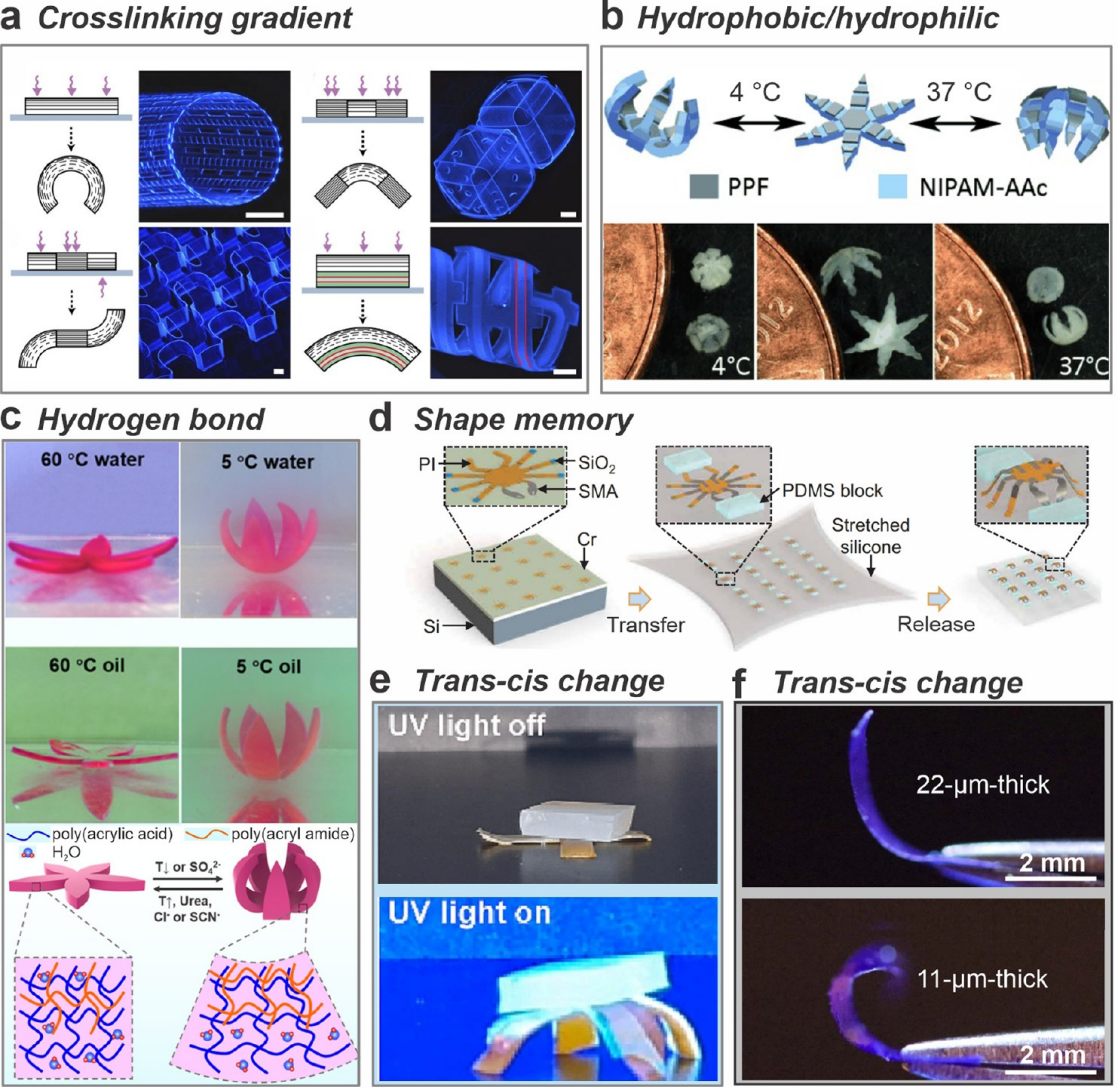
Shape-morphing mechanisms. (a) Schematics and fluorescence images of differentially photo-cross-linked and self-assembled SU8 geometries (scale bars are 250 μm). Reproduced with permission from ref (41). Copyright 2011, Springer Nature. (b) A drug-eluting gripper triggered by the temperature difference. Reproduced with permission from ref (42). Copyright 2014, Wiley-VCH. (c) Self-actuation behaviors of asymmetric PAAm/PAAc hydrogel flowers in water and oil bath. Adapted from ref (43). Copyright 2019, American Chemical Society. (d) Schematic illustration of the on-chip patterning and shape-morphing by a shape memory alloy. Adapted with permission from ref (44). Copyright 2022, The American Association for the Advancement of Science. (e) Weight lifting of the photodeformable bilayer films under UV light. Adapted with permission from ref (45). Copyright 2023, Elsevier. (f) The bending deformation of light-deformable polymer with different thicknesses. Adapted with permission from ref (46). Copyright 2023, Springer Nature.

Therefore, diversifying into alternative actuation mechanisms becomes imperative to propel further explorations in materials and hence advancements in micro-origami technology for high-performance microbatteries. In principle, all actuation mechanisms are possible to wind thin films up. Given the multiple layers within the energy storage system, a paramount consideration for actuation is to prevent any damage to the materials involved in energy storage; so, a preferred approach is a gradual and gentle shape-morphing process. Swelling is not limited to hydrogels. Figure 5a demonstrates the swelling gradient created by differential cross-linking of an SU8 film.^41^ The films can actuate through desolvation and resolvation in the solvent exchange between water and acetone, eventually forming self-assembled 3D structures due to the stress gradient in the films.^41^

In the case of thermal actuation, the difference in swelling is also an important driving force for the shape morphing. The most widely used polymer, poly(*N*-isopropylacrylamide) (pNIPAM), can be switched between hydrophilic and hydrophobic at programmed temperatures.^42^ As shown in Figure 5b, a small gripper using pNIPAM can be actuated by temperature.^42^ pNIPAM composited with PAAc is hydrophilic below 32 °C and swells in water. When the temperature is higher than 32 °C, extensive dehydration occurs, causing a transition to hydrophobic surfaces and hence returning to the original shape. Figure 5c exhibits a PAAm/PAAc (poly(acrylamide)/poly(acrylic acid)) bilayer hydrogel. The shape morphing is triggered at an upper critical solution temperature (UCST). When the temperature falls below the UCST, the PAAm/PAAc hydrogel contracts due to enhanced hydrogen bonding, whereas the PAAm layer keeps its swelling state. This differential swelling behavior causes the hydrogel flowers to close. Additionally, the hydrogel flowers are sensitive to urea and salts, as these substances alter the strength of the hydrogen bonds.^43^

Shape memory effects of materials also have been used as actuators. Figure 5d illustrates the on-chip integration of a shape memory alloy (nitinol).^44^ The shape programming process was done by a prestretched silicone elastomer. The release of the strain causes compressive stresses at the “legs”, converting the 2D design into a 3D architecture. An increase in temperature transitions nitinol from the martensitic to the austenitic phase, flattening the 3D structure into a 2D shape. Upon cooling, it reverts to martensitic, reducing force at the joints.^44^ Besides alloys, shape memory polymers, such as polyolefin,^47^ polystyrene,^48^ polybenzoxazines,^49^ and polyurethane,^50^ can be programmed to diverse shapes by entropic elasticity.^51^ In the shape programming process, the polymeric material reaches transition temperatures, such as the glass transition temperature or melting point.^51^ Subsequently, the material is temporarily deformed, then fixed in that state through controlled cooling. Finally, the polymeric material forms into the programmed shape when exposed to heat stimuli.^51^ For instance, shape changes through rolling, folding, twisting and wrinkling can be designed by using polyolefin and hyperelastic polymers.^47^

Light actuation has gained significant attention in recent years. Light-responsive materials can be divided into two categories: photothermal materials and photodeformable materials. Heat can be generated within the origami layer stack through energy conversion. Upon exposure to light, black phosphorus–carbon nanotube composites absorbed light energy and generated heat, causing thermal expansion of the polymer layer.^52^ Such a mechanism allowed the bilayer structure to bend. Other 2D materials, such as MXene,^53^ MoS_2_,^54^ and graphene,^55^ also exhibit a photothermal effect. By strategically combining various deformable materials, it is feasible to achieve diverse forms by light-induced actuation. Additionally, the utilization of laser-induced heating and momentum has proven to be an effective method in releasing thin films and shaping them into Swiss rolls, which facilitates the long-distance rolling of thin films and hence increases the mass loading of materials per unit area.^56^

Another approach to actuation involves the use of photodeformable materials, which consist of photoreactive molecules designed to absorb light at specific wavelengths and hence deform. Two photosensitive reactions for creating photodeformable materials are available: intramolecular photoisomerization and intermolecular photodimerization.^57^ Photoisomerization involves light-induced alterations of chemical structure such as trans–cis isomerization, enol-keto tautomerization, electrocyclic reaction, intramolecular hydrogen abstraction, and heterolytic cleavage.^57^ Photodimerization, however, modifies the molecular structure, prompting the movement of photoactive moieties and eventually yielding significant deformation and thermally stable products.^57^ Azobenzene, a widely studied photoresponsive molecule, exhibits reversible isomerization between its trans and cis conformations in liquid crystal networks. Under irradiation with 365 or 435 nm wavelengths, azobenzene shows a 27.8° shift in the C–N=N–C dihedral angle. This significant structural transformation facilitates the macroscopic shape alteration in polymers.^58^ The liquid crystal elastomers equipped with azobenzene moieties and boron ester bonds demonstrate anisotropic shape deformations upon UV light irradiation (Figure 5e).^45^Figure 5f demonstrates a roll-up process using a light-deformable material incorporating diarylethene microcrystals within a polyethylene terephthalate host matrix. The photoactuators demonstrated long-term reversibility due to photoisomerization based on the photoinduced ring opening and closing.^46^

The shape-morphing materials triggered by diverse stimuli lay the groundwork for the micro-origami process. However, alternative materials might have different thermal or chemical stability, imposing additional limitations in fabrication processes. Actuation materials must withstand mechanical stress during self-assembly and resist degradation in battery working conditions. These materials should offer precise and reliable actuation in response to actuation stimuli. By achieving high precision in both patterning materials and the shape-morphing process, a high yield of micro-origami devices can be guaranteed. As such, despite the relatively complex steps involved, the cost of micro-origami devices will not increase significantly when compared to the standard fabrication of thin-film devices.

### Unexplored Fields: Integration of Microbatteries

Unlike full-sized batteries such as the 18650-cell, which serve as universal components for devices ranging from hearing aids to electric vehicles, a single type of microbattery cannot function as a ubiquitous unit for diverse microelectronics. This limitation arises from the wide-ranging specifications of microelectronic systems, where different components demand varying power levels. For instance, sensors require low power, while actuators need high power for movement initiation. Even for the same component, such as CMOS, the power consumption varies significantly between idle and full-load modes. At small scales, a slight current change might exceed the limits of a single battery. Additionally, the up- and down-conversion of voltage from microbatteries leads to energy loss. Consequently, microbatteries are closely tied to their specific applications. From an application perspective, the integration of microbatteries with microelectronics is crucial, although this aspect has not been explored as extensively as the development of electrode materials for microbatteries. The same applies to the integration with energy harvesters like solar cells. The main concern of such integration is matching the output of the energy harvester and the charging requirement of microbatteries. Overcharging and fluctuation of input need to be avoided through delicate circuits that regulate currents and voltages.

**Figure 6:**
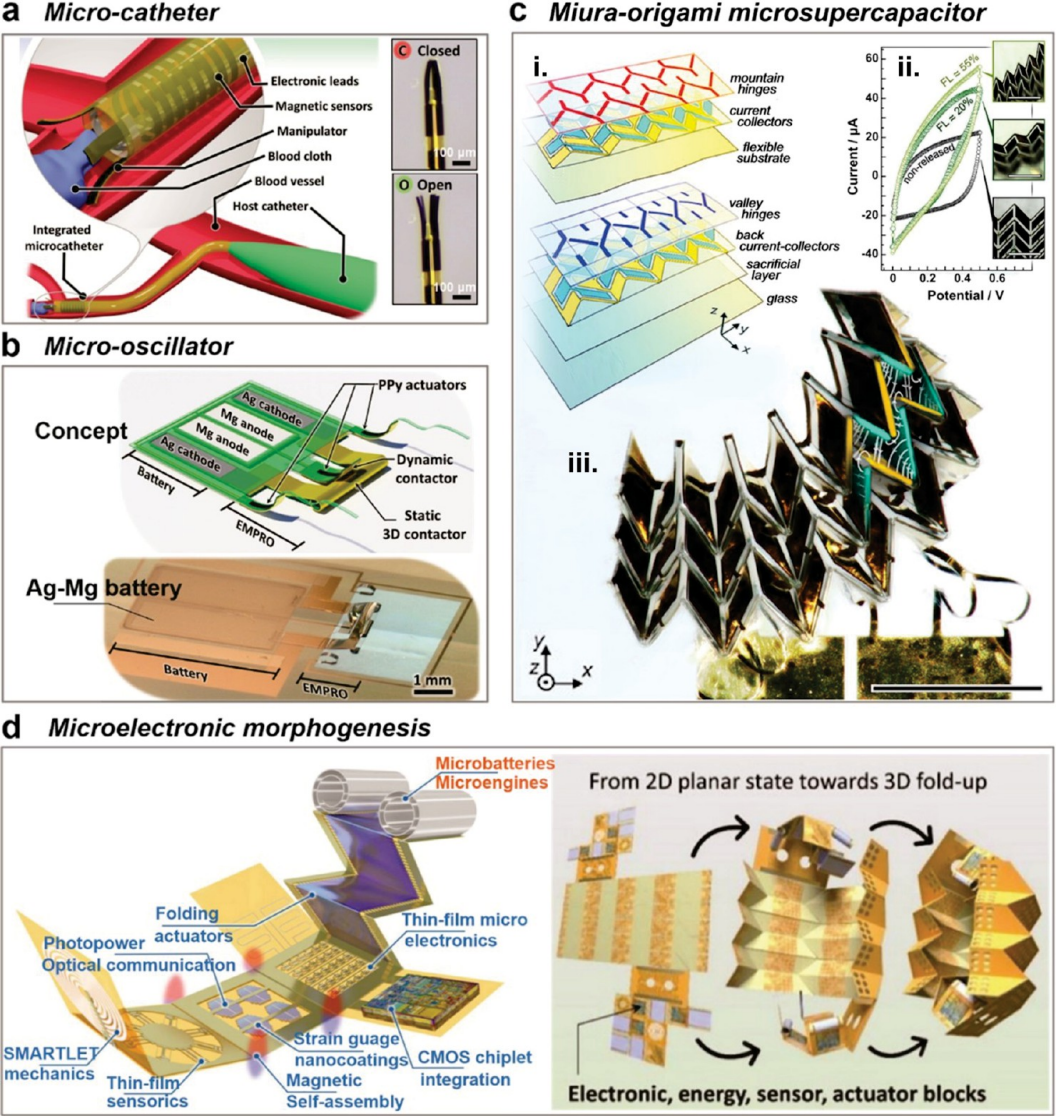
Integration of micro-origami energy storage into tiny devices. (a) The concept of the microcatheter deployed into a microscopic blood vessel (left) and the real microcatheter tip in its closed and open state (right). Adapted from ref (59). Available under a CC-BY 4.0 license. Copyright 2021 by the authors. (b) The concept and an image of the micro-oscillator integrated with Ag–Mg battery. Adapted from ref (60). Available under a CC-BY 4.0 license. Copyright 2021 by the authors. (c) Miura-origami microsupercapacitor based on chemo-mechanical hinges: exploded view of the microsupercapacitor component layers (i), device’s current–voltage cycling characteristics (ii), and multifocal microscopy image of the device (iii). Scale bars in subpanels: 500 μm. Adapted from ref (62). Available under a CC-BY 4.0 license. Copyright 2024 by the authors. (d) The concept of microelectronic morphogenesis toward complicated electronic systems by the micro-origami technology. Adapted from ref (63). Available under a CC BY-NC-ND 4.0 license. Copyright 2023 by the authors.

Take Swiss-roll microbatteries, for instance; they seamlessly complement tubular devices such as catheters. Figure 6a presents a microcatheter integrated with a gripper at one end, capable of capturing tiny particles in a confined space.^59^ The electronically actuated gripper, composed of a low-voltage actuator material (polypyrrole), switches within 0.8 V, a voltage range easily met by any microbattery. Integration concerns, however, center around the stability of microbatteries during bending, as catheters often navigate tortuous channels to reach target positions. Swimming microrobots, typically featuring a cylindrical structure to improve their movement in fluids, predominantly utilize electronically controlled actuation. Integrating a battery would enhance autonomy by freeing swimming microrobots from wires and external sources. Figure 6b provides another example: a micro-oscillator powered by an Ag–Mg primary battery for autonomous operation.^60^ The voltage oscillation results in the mechanical motion of the actuators. Replacing the primary battery with more energy-dense and rechargeable batteries can unleash the full potential of such oscillators.

Beyond the Swiss roll, a conventional battery architecture, there remains ample opportunity to explore alternative designs like folding structures of batteries. For instance, origami lithium-ion batteries have been created by folding the Miura-origami pattern.^61^ The Miura-origami consists of parallelogram faces linked by alternating mountain and valley creases, allowing for two types of folding: 45° Miura folding for compression in one direction, and 90° Miura folding for collapsibility in two directions.^61^ Despite the high deformability of the overall structure, the parallelogram faces stay undeformed. This is due to the nature of rigid origami, where the creases enable system-level deformability without significant strain on the substrate materials. Figure 6c exhibits another example: self-adaptive, ultraflexible 4D electronics using micro-origami folding, bringing this concept to the microscale level.^62^ The Miura-origami microsupercapacitor was developed based on patterned actuator hinges. The device’s multilayer composition was fabricated by the standard photolithography process. The current–voltage cycling characteristics of the device were acquired at distinct folding levels (FL), such as FL = 55%, 20%, and zero (nonreleased device). The power density of the Miura-origami micro supercapacitor exceeded 100 mW cm^–2^, which proves the potential of micro-origami folding for ultraflexible and autonomous microelectronic applications.

Miura-origami structures feature a unique folding pattern that allows them to compress or expand while preserving their structural integrity. Miura-origami avoids the strain caused by the curvature as most surfaces of a Miura-origami structure remain flat. Flat surfaces can load rigid materials and electronic components such as silicon chips, sensors, and energy harvesters. It is important to note that each configuration has unique advantages that should be considered based on specific application requirements. As illustrated in Figure 6d, the combination of Miura-origami and rolling structures is a possible way to realize the integration of “hard” components.^63^

Origami technologies are not limited to energy storage. One interesting application for origami structures is the use of cubic architectures for the development of 3-axial sensors.^10^ The sensors are located on all the faces of the cube, acquiring responses from the magnitude and direction of stimulus signals. The origami approach can also improve antenna performance. For instance, the frequency, radiation pattern, and polarization of the antennas depend on the length and direction in the plane of radiation and the orientation of the electric or magnetic current source, which are characteristics that can be controlled by folding and unfolding the origami platform.^64^ In addition, the origami process shrinks the antenna to a size suitable for microscale applications.^65^ A origami generated polyhedral structure could increase power transfer, allowing it to power an LED.^66^ By providing deformability, flexibility, and elasticity to systems, origami structures can improve energy-harvesting systems such as photovoltaic devices and triboelectric nanogenerators.^67,68^ These origami devices also offer viable processes to develop batteries and hence enrich technology pool for micro-origami energy storage.

## Conclusion

**Figure 7:**
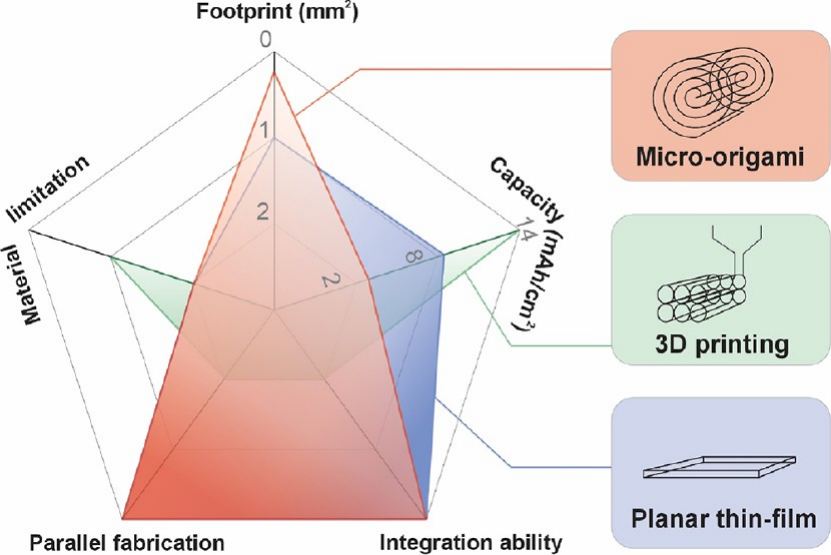
Comparison of micro-origami, 3D printing, and planar thin-film technologies in five dimensions: footprint, capacity, integration ability, parallel fabrication feasibility, and material limitations. Note that the boundary refers to the state-of-the-art values in each dimension.

Micro-origami stands as an emerging technology for creating tiny and energy-dense batteries and supercapacitors because the shape-morphing process aligns closely with proven approaches used in fabricating full-sized batteries and supercapacitors. As shown in Figure 7, the main advantage of state-of-the-art micro-origami technologies is the minimal footprint, reaching deep-submillimeter scales.^33^ Both planar thin-films and 3D printing are limited to above 1 mm^2^. However, as the micro-origami energy storage has not yet incorporated benchmark materials, the capacity is lower than that of thin-film and 3D-printed devices. It is worth noting that the 3D printing technique can achieve a high capacity of over 10 mAh cm^–2^,^69^ yet its footprint is usually large. Should micro-origami technology advance to become a versatile tool capable of morphing any thin-film device, it could significantly enhance capacity limits by enabling the multiplication of thin-film system values several times over. In addition to the energy storage performance, the integration ability, parallel fabrication feasibility and material limitations are crucial factors for practical applications. Thin-film and micro-origami technologies largely rely on on-chip fabrication tools; they are inherently suitable for high-degree integration (e.g., monolithic integration) with other on-chip devices. Meanwhile, on-chip fabrication processes allow for parallel fabrication. Therefore, the integration ability and parallel fabrication feasibility of thin-film and micro-origami devices are comparable. However, these aspects are considered shortfalls for 3D printing, which typically requires an independent manufacturing process. Conversely, the separate manufacturing approach of 3D printing allows for a broader selection of materials. The constraints of printable slurry formulations are less restrictive than the intrinsic limitations associated with the deposition methods employed in micro-origami and thin-film device fabrication.

It is clear that micro-origami energy storage is not a solution for all purposes. They will find their applications where space is at a premium. For micro-origami technology to take root in microscale energy storage, it is crucial to emphasize the engineering of materials, ensuring compatibility with modern microfabrication processes, and assessing feasibility for integration into specific applications. Gaining a deeper understanding of material-device interactions, diverse application requirements, and the challenges and limitations of different components is imperative.
